# Molecular mechanisms of dendritic cell migration in immunity and cancer

**DOI:** 10.1007/s00430-020-00680-4

**Published:** 2020-05-25

**Authors:** Charlotte M. de Winde, Clare Munday, Sophie E. Acton

**Affiliations:** Stromal Immunology Group, MRC Laboratory for Molecular Cell Biology, University College London, Gower Street, London, WC1E 6BT UK

**Keywords:** Dendritic cell, Cell migration, Actin cytoskeleton, Tetraspanin, Integrin, Lectin

## Abstract

Dendritic cells (DCs) are a heterogeneous population of antigen-presenting cells that act to bridge innate and adaptive immunity. DCs are critical in mounting effective immune responses to tissue damage, pathogens and cancer. Immature DCs continuously sample tissues and engulf antigens via endocytic pathways such as phagocytosis or macropinocytosis, which result in DC activation. Activated DCs undergo a maturation process by downregulating endocytosis and upregulating surface proteins controlling migration to lymphoid tissues where DC-mediated antigen presentation initiates adaptive immune responses. To traffic to lymphoid tissues, DCs must adapt their motility mechanisms to migrate within a wide variety of tissue types and cross barriers to enter lymphatics. All steps of DC migration involve cell–cell or cell–substrate interactions. This review discusses DC migration mechanisms in immunity and cancer with a focus on the role of cytoskeletal processes and cell surface proteins, including integrins, lectins and tetraspanins. Understanding the adapting molecular mechanisms controlling DC migration in immunity provides the basis for therapeutic interventions to dampen immune activation in autoimmunity, or to improve anti-tumour immune responses.

## Introduction

Dendritic cells (DCs) are professional antigen-presenting cells central to the induction of adaptive immune responses and to the promotion of self-tolerance. In 1973, Steinman and Cohn were the first to isolate these cells from murine peripheral lymphoid organs and named them after their constantly extending and retracting fine dendritic cell processes [1]. Steinman was later awarded a Nobel Prize for this discovery and his subsequent work in determining the role of DCs in adaptive immunity [2]. DCs are a highly heterogeneous population of cells, which have historically been categorised according to their phenotype, function or location. However, DCs have more recently been defined as a haematopoietic lineage in their own right [3]. DCs originate from precursor cells, such as monocytes and pre-DCs, in the bone marrow. Upon leaving the bone marrow, these precursors migrate to peripheral tissues and secondary lymphoid organs via blood vessels. There are five classic subsets of DCs defined in humans—conventional DCs type 1 and 2 (cDC1 and cDC2), plasmacytoid DCs (pDC), monocyte-derived DCs (moDC), and Langerhans cells [4]—and there are equivalent DC subsets in mice [5] (Table 1). Recently, this classification has been revisited, and additional DC subsets are defined [6, 7]. Each subset resides in a different niche throughout the body and has a specific role in the immune response [8, 9] (Table 1).

**Table 1:** Overview of classic definition of human, and equivalent mouse, dendritic cell subsets

Human DC subset	Mouse DC subset	Development and function	Surface markers	Tetraspanin surface expression
Classical DC type 1 (cDC1)	CD8α+ DC	Bone marrow-derived, myeloid origin Able to cross-present antigens to CD8+ T cells via MHC class I Promote Th1 and natural killer cell responses Involved in immunity against intracellular pathogens, viruses, and cancer	CD13 CD103 CD141 CLEC9A XCR1	CD9 CD37 CD53 CD81^hi^ CD82 CD151 Tspan31
Classical DC type 2 (cDC2)	CD4+ DC	Bone marrow-derived, myeloid origin Able to activate Th1, Th2, Th17, and CD8+ T cells Involved in immunity against bacteria and fungi	CD1c CD11b CD11c SIRPα	CD9 CD37 CD53 CD81^hi^ CD82 CD151 Tspan31
Plasmacytoid DC (pDC)	Plasmacytoid DC (pDC)	Bone marrow-derived, lymphoid origin Secrete large amounts of IFN type 1 in response to TLR7/9 activation Mainly involved in anti-viral immunity	CD123 CD303/CLEC-4C CD304/NRP1	CD9^lo^ or CD9^hi^ CD37 CD53^hi^ CD81^lo^ or CD81^hi^ CD82^lo^ CD151^lo^ Tspan31
Langerhans cell	Langerhans cell	Derived from erythromyeloid progenitors found in the foetal liver Reside within epithelial layers Capable to self-renew	CD1a CD207/Langerin E-Cadherin	Not studied to date
Monocyte-derived DC (moDC)	Bone marrow-derived DC (BMDC)	Bone marrow-derived, myeloid origin Present in tissues during steady state, but also expand populations of tissue-resident DCs during inflammation	CD1a CD1c CD11c CCR2	Not studied to date

Each human DC subset has a distinct development and function, and is identified by the expression of different surface markers, and tetraspanin expression pattern [4, 5, 89, 90, 165, 166]

Dendritic cells are challenged with trafficking enormous distances throughout their life cycle, exiting the bone marrow, and entering and seeding all organs and tissues, then upon activation migrating to lymphoid tissues to initiate adaptive immunity. Within the tissues, immature DCs act as sentinels, and alert to signs of tissue damage or infection [10–12]. Importantly, DCs play a key role in initiating an anti-cancer immune response [13, 14] as they can also detect tumour antigens produced by cancer cells, such as mutated or aberrantly expressed proteins [15]. Uptake of foreign antigen induces DC maturation which enables them to migrate to the lymph nodes via lymphatic vessels [16, 17]. Once activated, DCs migrate to enter lymphatic vessels to traffic to lymphoid tissues where they must identify and activate their cognate T cells to initiate adaptive immunity [18, 19].

En route through the body, DCs traverse a wide range of diverse tissue environments and are required to cross barriers between different tissues and vessels to carry out their function. Immature DCs first scan peripheral tissues, before migrating through the lymphatics and, finally, within secondary lymphoid tissues such as lymph nodes. This wide range of microenvironments requires DCs to deploy different migration mechanisms, controlled by a diverse range of soluble and membrane-bound proteins. In this review, we discuss the molecular mechanisms involved in DC migration through these diverse environments focussing on the roles of the actin cytoskeleton and membrane proteins, including adhesion molecules and tetraspanins. Tetraspanins, a family of transmembrane proteins, interact with membrane and intracellular proteins to organise the plasma membrane into tetraspanin-enriched microdomains, which facilitate cell–cell interactions and effective downstream signalling [20–22]. Tetraspanins are expressed on DCs [23], and several tetraspanins have been implicated in controlling DC migration through interactions with C-type lectin receptors, integrins or small GTPases [24–27]. Finally, we explore DC migration in cancer. Understanding which migratory mechanisms may be impaired can aid in the development of anti-cancer therapies aiming to restore DC function and enhance presentation of tumour antigens.

## Seeding of peripheral tissues

It is believed that the final differentiation into immature DCs happens upon leaving the blood and entering the tissues [28–31]. Similar to other leukocytes, extravasation of DC precursors is thought to occur in three main steps and involves coordinated signalling through cytokines, selectins and integrins [32–34], potentially mediated by tetraspanin proteins [26, 27]. First, tethering occurs which causes the cells to slow down and roll along the endothelium. L-selectin expressed on circulating (pre-)DCs, and E- and P-selectin on activated endothelial cells are required for this process [35]. L-selectin or P-selectin deficient mice showed impaired leukocyte rolling and homing of lymphoid and peripheral tissues [36, 37]. P-selectin-dependent rolling is decreased in the absence of tetraspanin CD63 [38]. This is explained by decreased surface expression and clustering of P-selectin in CD63-deficient endothelial cells, indicating tetraspanin CD63 as an important partner protein for P-selectin [38].

Secondly, DC precursor cells undergo adhesion resulting in arrest of movement. This is initiated by binding of chemoattractants expressed by blood endothelium to CX3CR1 on pre-DCs [29, 39]. This causes a conformational change and activation of α4β1 and β2 integrins on the DC precursor cells, which will subsequently bind their ligands such as ICAM-1/2, VCAM-1, and MAdCAM-1 expressed on blood endothelial cells [32]. This causes firm adhesion and arrest of the cells, and finally, exit from the blood vessels via diapedesis [32]. Five tetraspanin family members, CD9, CD37, CD53, CD81, CD82, and CD151, are involved in the regulation of α4β1 and/or β2 integrins on several leukocyte types [40–49], but their role on rolling and transmigration of DC precursor cells into peripheral tissues is not explored. *Cd37* and *Cd81* knock-out mice have a normal immune system development [50, 51], and it is therefore not expected that these tetraspanins are required for homing of DC precursor cells to peripheral tissues. However, as some tetraspanin proteins are genetically similar [52], compensation mechanisms by other tetraspanins in this process cannot be excluded.

## Activation of dendritic cells by pathogens and danger signals

Immature DCs are activated upon recognising pathogen-associated or damage-associated molecular patterns (PAMPs or DAMPs) via pattern recognition receptors (PRRs) [53, 54]. PAMPs are mostly derived from pathogens and include molecular motifs, such as bacterial lipopolysaccharide (LPS) or nucleic acids [55]. In contrast, DAMPs are danger signals, many of which are aberrantly expressed self-molecules, produced upon stress or injury, for example dying cells, necrosis or cancer [53, 56]. PRRs are found both on and within many immune cells allowing detection of both extracellular and intracellular danger signals, respectively [55]. One important subgroup of PRRs is the Toll-like receptors (TLRs), a protein family composed of twelve different receptors expressed on leukocytes and stromal cells, which are able to detect both DAMPs and PAMPs [53, 57]. TLR stimulation initiates a signalling cascade resulting in activation of transcription factors, including NF-κB [58]. NF-κB is known to promote the expression of pro-inflammatory cytokines, which further stimulates an immune response [59]. In some cell lines, NF-κB has been shown to upregulate expression of the chemokine receptor CCR7, a critical signalling molecule for the homing of DCs to the lymphoid tissues [58, 60]. Additionally, others have suggested that inflammatory cytokines produced in response to TLR stimulation, such as tumour necrosis factor alpha (TNFα), may activate DCs in certain tissues [61, 62]. However, in vivo experimental evidence has shown that these mediators in isolation are not sufficient to induce full activation of DCs within secondary lymphoid tissues [63].

One common DAMP molecule, released upon cellular damage, is adenosine triphosphate (ATP), which is normally only present at very low levels within tissues. DCs sense high levels of extracellular ATP through P2X_7_ purinergic receptors, which triggers fast migration of DCs [64]. ATP-dependent activation of P2X_7_ instigates the opening of pannexin 1 (Panx1) membrane channels in the plasma membrane. This permits the release of intracellular ATP, which is able to act in an autocrine fashion to perpetuate fast migration. As well as stimulating Panx1 channels, P2X_7_ activation also allows entry of extracellular calcium into the DC [64], which may directly or indirectly stimulate reorganisation of the actin cytoskeleton. This happens particularly at the cell rear where it causes the formation of a large pool of F-actin critical for fast DC migration [64].

## Migration of dendritic cells within peripheral tissues

A population of immature DCs resides in every tissue of the body. They constantly patrol and sample for antigens, which are engulfed by receptor-mediated phagocytosis or non-specific macropinocytosis [65, 66]. Immature DCs prioritise these endocytic processes to facilitate their immune sentinel function. Conversely, immature DCs have a limited migratory capacity and there is low expression of molecules required for antigen presentation [67]. Immature Langerhans cells reside within epithelial layers and constitute one of the first lines of immunological defence against pathogens [68]. Lack of migratory activity allows them to form a dense network across the interfaces between tissues and the external environment. In this sessile state, Langerhans cells repeatedly extend and retract protrusions into intercellular spaces and also between epidermal cells. This behaviour enables sampling of a large area of the epidermis whilst remaining stationary [69, 70]. Other immature DC subsets do not tend to remain sessile, although their movement is still limited until they undergo maturation. Once DCs recognise a potential threat, they switch their behaviour away from endocytosis and towards migration. To move through tissues, DCs form actin-rich protrusions at the leading edge of the cell, which is accompanied by passive movement at the trailing edge, allowing the so-called “flowing” of the cell [71]. Conversely, “squeezing” of the cell, allowing forward movement of the nucleus, is facilitated by the motor protein myosin II, resulting in contraction at the cell rear. DCs have been described to rapidly move in an amoeboid-like fashion, using high actomyosin contractility through the cell cortex to constantly alter their shape [72–75]. This mode of motility is independent of integrins as ablation of integrin function by deletion of all integrin heterodimers and Talin, responsible for integrin activation, did not affect DC migration in three-dimensional (3D) environments or in vivo [71].

The discovery that DC migration within tissues is integrin-independent brought into question the previous assumptions regarding the role of mechanical forces in migration. Adhesive cells are known to exert large forces upon the surfaces on which they migrate, decreasing their sensitivity to small forces [76–78]. Conversely, it has been shown that cells migrating independently of adhesion molecules exert significantly smaller forces on the substratum [78, 79], suggesting that migrating DCs may indeed be sensitive to small forces. Hydraulic resistance, created by displacement of fluid as cells move through tissue, coupled with geometric confinement is the main factors which restrict DC movement within tissues. However, immature DCs have decreased sensitivity to hydraulic resistance as a consequence of their constitutive ability to engulf extracellular fluid non-specifically by macropinocytosis [65, 78]. Inhibition of macropinocytosis was shown to restore barotaxis (i.e. following paths of least resistance) in immature DCs [78]. Although the main function of macropinocytosis is antigen uptake, its ability to attenuate hydraulic resistance and thus overcome barotaxis is very useful. This permits immature DCs to patrol tissues more thoroughly, particularly through (parts of) tissues with high hydraulic resistance which may otherwise be inaccessible. Potentially, DCs may be able to increase macropinocytosis in response to external stimuli, like increased volume of extracellular fluid, facilitating effective sentinel activity during inflammation [78].

Whilst patrolling tissues, immature DCs tend to move at fluctuating speeds [80]. This can be explained by the antagonistic effects of myosin IIA in fast cell migration versus macropinocytosis. During phases of slow movement, high levels of myosin IIA are observed at the front of DCs [81]. Further analysis using a microfluidic device capable of separately altering myosin IIA activity at the front and back of the cell revealed that the slow movement was caused by myosin IIA activity at the cell front. This suggests that anterior accumulation of myosin IIA slows down DC movement by disrupting the normal front-to-back myosin gradient within the cell. Enrichment of myosin IIA at the DC front is controlled by the MHC class II-associated protein invariant chain (CD74) [81]. The localisation of myosin at the DC front is, furthermore, important for macropinocytosis, as both myosin II-deficient and CD74-deficient DCs showed less efficient formation and rearward intracellular transport of macropinosomes [81]. Another putative reason for the variable speeds observed within immature DCs is the regulation of filamentous actin (F-actin) within the cell. During slow movement, F-actin is accumulated at the cell front of immature DCs [82]. Conversely, during phases of faster movement, nucleation of a small pool of F-actin at the cell rear promotes cell migration. It was suggested that the protein complex Arp2/3, known to nucleate branched actin [83], may be responsible for the accumulation of F-actin at the cell front. In agreement with this hypothesis, inhibition of Arp2/3 in immature DCs resulted in a reduction of F-actin at the front of the cell. Furthermore, the Arp2/3 complex, activated by the small GTPase Cdc42, was shown to be specifically localised around macropinosomes at the front of the cell. Knock-out of the Arp2/3 complex protein ARPC2 further showed the importance of the Arp2/3 complex in effective formation of macropinosomes [82]. Thus, fluctuating speeds of immature DCs facilitate effective endocytosis during space exploration by immature DCs. Both myosin IIA and the Arp2/3 complex play important roles in macropinosome formation required for efficient antigen uptake by immature DCs.

## Directional migration of dendritic cells towards lymphatics

Activation of DCs by PRR stimulation causes DC maturation [54]. During the maturation process, DCs downregulate processes linked to their sentinel function. Macropinocytosis is decreased by downregulation of Cdc42 and a reduction of Arp2/3 levels within the cell [82]. In response to decreased macropinocytosis, DC sensitivity to hydraulic resistance is increased, and thus, they begin to undergo barotaxis. Neutrophils have also been shown to exhibit barotaxis in confinement, favouring the path of least resistance [84]. Barotactic movement enables the activated DCs to take the most direct route to the nearest lymph vessel, as they avoid long routes and dead ends which have a higher resistance [78]. Furthermore, upon maturation, DCs increase the expression of cell surface molecules related to antigen presentation and directional migration (Table 2) [85–88]. Differential expression of tetraspanins CD9 and CD81 on human pDCs (Table 1) defines subsets with different localisation and function [89, 90]. However, it is currently unknown if DC activation and maturation changes tetraspanin expression, and as such controls DC migration.

**Table 2:** Key cell surface proteins and associated tetraspanins in dendritic cell immune function [85–88, 91, 100, 126, 167–169]

	Maturation markers	Function	Tetraspanin interaction^a^
Adhesion	Semaphorin 7A (Sema7A)	Stimulate moDC migration by reducing adhesion and promoting protrusion formation	Unknown
	Lymphocyte function-associated antigen 1 (LFA1)	Integrin able to regulate the duration of contact between DCs and naïve T cells during antigen presentation	CD9 [41], CD53 [49, 170, 171], CD81 [172], CD82 [48]
Antigen cross-presentation	Major histocompatibility complex I (MHC-I)	Allow presentation of intracellular protein-derived peptides to CD8+ T cells	CD53 [49, 173], CD82 [174]
	Major histocompatibility complex II (MHC-II)	Allow presentation of extracellular protein-derived peptides to CD4+ T cells	CD9, CD37, CD53, CD63^b^, CD81, CD82 [49, 173, 175–178]
Co-stimulation	CD40	Receptor involved in further DC activation	Unknown
	CD80, CD83, CD86	Co-stimulatory surface proteins needed for T-cell activation	CD151 [179]^c^
Migration	Chemokine receptor 7 (CCR7)	Chemokine receptor required for DC migration to the LN	Unknown
	C-type lectin-like receptor 2 (CLEC-2)	Interaction with podoplanin, a glycoprotein expressed on the surface of LECs and FRCs	CD37 [127]

^a^Tetraspanin interaction with these molecules has not all been reported on DCs

^b^CD63 is localised intracellularly

^c^CD151 on DCs controls co-stimulation of T cells during antigen presentation via MHC-II, but the exact mechanism is unknown

Expression of the G-protein coupled chemokine receptor CCR7 is required for DC migration through the lymphatic system [91]. The chemokines CCL19 and CCL21 are both ligands of CCR7, but CCL21 is thought to be the chemokine critical for DC migration [92, 93], whereas CCL19 plays a less significant role [94]. Lymphatic endothelial cells (LECs) are constitutively expressing CCL21 allowing chemotaxis of DCs during steady state [92, 95]. Upon inflammation, CCL21 expression is upregulated on LECs following the detection of pro-inflammatory cytokines, like TNFα, which facilitates increased haptotaxis of DCs towards the nearest lymphatic vessel [95]. Haptotaxis is a form of directed cell movement along immobilized gradients of adhesion cues or chemokines [92, 96]. The highly positively charged C-terminus of CCL21 can bind to heparin sulphates present on cell surfaces and within the extracellular matrix, thus forming a long-lasting local gradient of CCL21 on LECs [92]. The gradient starts approximately 90 μm from the lymphatic vessel, which coincides with the distance at which DCs shift from random to highly directional movement [92]. Oligomerisation of CCR7 on the cell surface of DCs, induced by the inflammatory mediator prostaglandin E2 (PGE_2_), has been postulated to play a role in efficient migration of some DC subsets towards CCL21 [97]. CCR7 oligomerisation allows binding and activation of Src family kinases, initiating Src signalling pathways in addition to G-protein coupled receptor signalling from CCR7. Phosphorylation of oligomeric CCR7 by Src at a tyrosine residue creates a binding site for further signalling molecules containing SH2-domains, which is important for efficient cell migration towards CCL21 [97]. The gap junction protein connexin43 (Cx43) expressed in cDCs has also been identified as a potential player in DC migration towards CCL21 [98]. In vitro studies using bone marrow-derived DCs (BMDCs) from mice with reduced Cx43 expression revealed defective migration towards CCL21. Moreover, reduced cDC migration to the lymph node in vivo was observed in mice expressing a truncated form of Cx43 [98]. Although there was no direct connection defined between Cx43 and the directional movement of DCs, it has previously been noted that connexin interacts with c-Src kinase involved in CCL21-directed movement [97–99]. Human monocyte-derived mature DCs highly express the GPI-anchored protein semaphorin 7A (SEMA7A) which has been shown to promote chemokine-driven DC migration [100]. Ex vivo assays with LPS-stimulated BMDCs from *Sema7A* knock-out mice showed a reduced capacity to migrate towards CCL21, despite expressing similar surface levels of CCR7. However, when replicated in vivo, these results were not demonstrated to be significant, and it was suggested that this was due to the complicated multi-step migration that occurs in vivo. Interestingly, when using a collagen matrix to simulate the complex tissue environment, migration of mature DCs with reduced SEMA7A expression (SEMA7A-KD) was more significantly decreased. These SEMA7A-KD DCs also lacked the ability to efficiently form actin-rich protrusions causing a slower migration through the 3D environment and were more adhesive. This suggests that, SEMA7A at least partially, controls migration by reducing cell adhesion and promoting protrusion formation [100].

DC maturation induces cytoskeletal changes, which optimise DC motility to permit fast migration [82]. In contrast to immature DCs, the main location of F-actin in mature DCs is within the cell cortex at the rear of the cell [82]. The Formin protein family member mDia1, activated by the small GTPase RhoA, was shown to be critical in maintaining F-actin at the cell rear, thereby ensuring fast migration. Moreover, experiments involving mDia1 knock-out DCs, suggested its involvement in facilitating chemotaxis of mature DCs towards CCL21 [82].

## Entry into lymphatics

To reach the lymph nodes, DCs must enter the afferent lymphatic vessels by a process known as intravasation. DCs tend to enter the lymphatics at the blind-ended initial lymphatics, called lymphatic capillaries [101, 102]. The first step in intravasation involves crossing the extracellular matrix barrier of the basement membrane surrounding the lymphatic vessel. This basement membrane has a discontinuous structure, and intravasating DCs scan for gaps to traverse into the lymphatics [102]. DCs extend a cell protrusion into the opening, before contracting the cell rear to squeeze through the extracellular matrix barrier [102]. Next, they must cross the monolayer of LECs. Between oak-leaf shaped LECs that align the lymphatic capillaries, there are specialised junctions, containing a button-like distribution of adhesion molecules [101]. Similar to other endothelial cell junctions, the adhesion molecules expressed include tight junction proteins and VE-cadherin. However, these specialised junctions also specifically express high levels of the lymphatic vessel endothelial protein (LYVE-1), which acts as receptor for hyaluronic acid (HA) [103]. HA is found to be expressed on the surface of DCs, and HA binding is increased upon DC maturation [104, 105]. DCs attach to LECs via interactions between HA and LYVE-1^+^ transmigratory cups, which extend from the LECs and engulf the DC, facilitating entry into the lymphatic capillary [105]. Disruption of the interaction between LYVE-1 and HA using monoclonal antibodies resulted in reduced entry of DCs in the lymphatic vessels [105], suggesting that this molecular interaction is critical for transmigration.

As discussed above, DC migration through a 3D environment (i.e. the tissue) occurs independently of integrins [71]. However, upon inflammation, lymphatic endothelium upregulates expression of integrin ligands (e.g. ICAM1 and VCAM1), promoting adhesion-mediated DC transmigration [95, 106, 107]. In the presence of the pro-inflammatory cytokine TNFα, blocking of β2 integrin using monoclonal antibodies resulted in a reduction in DC transmigration [95]. The adhesion molecule L1 (also known as L1CAM or CD171) is involved in neuronal cell migration and cell–cell adhesion by intercellular binding to L1 or integrins [108]. L1 is also expressed on the surface of some DC subsets, including Langerhans cells [109, 110]. L1-negative DCs show reduced adhesion to the endothelium and impaired transmigratory capacity across the lymphatic endothelium [110], indicating L1 as an important player in DC intravasation.

CCL21 is also thought to play a role in DC intravasation as it stimulates DC migration across the endothelium in vitro [95]. CCL21 has been observed within intracellular vesicles and the trans-Golgi network within LECs [111]. Molecular interactions between DCs and LECs, and mechanical forces exerted by DCs onto the LECs, increase intracellular calcium concentrations, which acts as a signal for secretion of intracellular CCL21 [111], and may stimulate the DC to pass through the LEC monolayer. In addition, the molecular and physical signals acting on the LEC may combine to propagate entry into the lymphatics [111]. Semaphorin 3A, expressed on LECs, may also be involved in the guidance of DCs into the lymphatics [112]. Semaphorin 3A is able to promote actomyosin contraction of the cell rear via its receptor components Plexin-A1 and Neuropilin-1 (NRP1), found at the trailing edge of the cell. This contraction facilitates squeezing of the DC through the lymphatic endothelium, and mice with deletion of the Plexin-A1 gene *Plxna1* were shown to have reduced migration to the lymph nodes [112].

## Migration through lymphatics and entry into the lymph nodes

Once DCs have entered lymphatics, they require 24–72 h to reach the draining lymph nodes. Within the lymphatics, DCs move slowly along the vessel wall. Passive movement along with flowing lymph may play a role, but the hydrodynamic forces within the slow-moving lymph of the capillaries are suspected to be insufficient [113]. Within the lymphatics, similar to interstitial movement, DCs crawl by active extension of protrusions at the cell front [114]. Rho-associated protein kinase (ROCK)-driven contractility has been suggested to play a role in intralymphatic DC migration [115–117]. Inhibition of ROCK in steady state was shown to slightly decrease intralymphatic migration, but during inflammation, the contribution of ROCK activity was much more significant [117]. Furthermore, intralymphatic migration of DCs towards the lymph nodes is thought to be reliant on interactions between CCL21 and CCR7 [91, 113]. CCL21 is present both within the lymph and on the luminal surface of LECs, and forms a functional gradient within the lymphatic capillaries [113]. Although blockade of either CCR7 or CCL21 did not affect the movement of DCs within the lymphatics, it did severely impact migration towards the lymph node [113], suggesting that the CCL21 gradient is required for directional DC migration towards the lymph node. After leaving the lymphatic capillaries, DCs enter the large collecting lymph vessels. Lymph is able to flow faster in these vessels due to the presence of contracting lymphatic muscle cells surrounding the vessels, and intraluminal valves which prevent backflow of the lymph [118]. This higher speed allows DCs to passively move with the lymph up to speeds of around 1200 µm/min [114].

Upon entry into the lymph node, DCs migrate through the floor of the subcapsular sinus towards the paracortex, which is composed of T lymphocytes and fibroblastic reticular cells (FRCs) [119]. FRCs are specialised fibroblasts that produce and enwrap reticular fibres, made of collagen fibrils and other extracellular matrix components, which together form the conduit network allowing lymph to flow through the lymph node. FRCs form a 3D network within the lymph node, which serves as a scaffold for immune cell migration [120–122]. FRCs also express both CCL19 and CCL21, thereby contributing to the chemokine gradient guiding DCs towards the lymph node paracortex to activate T cells [123]. In addition to CCR7/CCL21 interaction, FRCs and LECs express the glycoprotein podoplanin [124], the ligand for C-type lectin-like receptor 2 (CLEC-2) upregulated on DCs during maturation [125, 126]. Interactions between podoplanin and CLEC-2 play a role in DC migration through the lymphatics and within the lymph node [126, 127]. Deletion of CLEC-2 from DCs or podoplanin from FRCs impairs DC migration along these stromal cell scaffolds [126]. Interaction of podoplanin with CLEC-2^+^ DCs induces the formation of highly branched protrusions with an accumulation of F-actin at the tips, whereas CLEC-2-deleted DCs were unable to form protrusions [126]. Interestingly, podoplanin and CCL21 interact with each other on LECs [128], but a role of this interaction in DC migration has not been directly addressed. Loss of tetraspanin CD9, a partner protein of podoplanin [129], or tetraspanin CD82 causes a decrease in podoplanin surface expression [130, 131]. Although DCs could still bind to and interact with CD9-deficient FRCs [130], the role of tetraspanins in CLEC-2/podoplanin-dependent DC migration has not been studied.

CLEC-2 activation initiates a signalling cascade via spleen tyrosine kinase (Syk) [132]. Podoplanin binding to CLEC-2 results in Syk-dependent activation of Vav, which, in turn, activates the RhoGTPase Rac1 driving formation of actin-rich protrusions. Indeed, increased Rac1 activity was observed upon CLEC-2 activation by recombinant podoplanin [126]. Simultaneously, RhoA activity was decreased, which reduced the level of phosphorylated myosin light chain (pMLC) within the cell, resulting in decreased actomyosin contractility enabling DCs to spread along the FRC surface [126]. Tetraspanin CD37 directly interacts with CLEC-2, and expression of CD37 is required for clustering of CLEC-2 upon podoplanin binding [127]. DCs from *Cd37* knock-out mice, similar to CLEC-2-deficient DCs, show impaired protrusion formation upon stimulation with recombinant podoplanin, and reduced migratory capacity [127, 133]. This indicates that CD37-enriched microdomains facilitate localisation of CLEC-2 and downstream signalling activation.

Tetraspanin CD81 can directly interact with Rac1 GTPase [134], and is required for Rac1 and integrin localisation at the leading edge of DCs [135]. CD81 knock-down DCs were unable to form lamellipodia protrusions, which significantly reduced their migratory capacity in the presence of a CCL19 gradient in a 2D environment. Loss of CD81 expression did not affect integrin-independent DC migration in a 3D environment [135], indicating that CD81 only controls adhesion-mediated cell migration by coupling integrin function to the actin cytoskeleton. Conversely, tetraspanin CD82 is upregulated in activated DCs, and decreases DC migration by reducing activation of RhoA [136]. This results in cytoskeletal rearrangements allowing DC spreading, which facilitates the formation of stable interactions between DCs and T cells to present antigens and effectively induce an immune response [136].

## Dendritic cell migration in cancer

Tumour cells have often lost the ability to undergo programmed cell death [137], so activation of the immune system is a powerful therapeutic strategy to both eradicate tumour cells and prevent further growth at metastatic sites [13, 14]. The cellular composition of the tumour microenvironment is critical to tumour growth and in determining response to therapy [138]. Besides tumour cells, the tumour microenvironment consists of innate and adaptive immune cells, and other stromal cells such as fibroblasts [139]. Immune cell types within the tumour microenvironment can promote or inhibit cancer progression, but pDCs play a dual role [140]. On one hand, pDCs suppress anti-tumour immunity by activating regulatory T cells within the tumour microenvironment [141, 142]. As such, recruitment and presence of pDCs in tumours are often correlated with poor prognosis [141, 142]. On the other hand, pDCs activated with tumour antigens, either ex vivo [143, 144] or via in vivo targeting [145], drive a potent cytotoxic CD8^+^ T cell response against the tumour. This double pDC function in cancer can potentially be explained by functional differences in pDC subsets, which are characterized by differential expression of tetraspanin CD9 and CD81 [89, 90]. CD9^−^CD81^+^ pDCs induce regulatory T cells and are immunosuppressive, whereas CD9^+^CD81^−^ pDCs secrete type I interferon (IFNα) and drive anti-tumour immunity via activation of cytotoxic CD8^+^ T cells [89, 90]. Expression of these two tetraspanins can be used to characterize pDC subsets in the tumour microenvironment, and to enrich for pDCs with immune promoting capacity in therapeutic DC vaccinations [146].

cDCs, and in particular cDC1s, have been associated with immune destruction of cancers [147, 148]. The anti-cancer functions of cDC1s are associated with their ability to take up and cross-present tumour antigens via MHC class I molecules to naïve CD8^+^ T cells in the tumour-draining lymph node [149]. Upon activation, cytotoxic CD8^+^ T cells are able to migrate to the tumour and kill cancer cells. cDC1 cells are further able to assist CD8^+^ T cells by producing large amounts of interleukin-12 (IL-12), a cytokine which is known to support the cytotoxic effector function of CD8^+^ T cells [150]. Although cDC1s are seldom found in the tumour microenvironment, their presence in melanoma is linked to increased T cell infiltration [151]. Therefore, it was suggested that if levels of cDC1 in the tumour microenvironment could be increased, it may increase the efficacy of immunotherapy.

One recent line of research suggests that the inflammatory environment of tumours can be targeted therapeutically to alter DC infiltration and enhance anti-tumour T cell responses [147]. Prostaglandin E2 (PGE_2_) is an eicosanoid secreted by many cells throughout the body, particularly during cell death [152]. PGE_2_ has been linked to the promotion of cancer by causing immunosuppression, as well as supporting processes such as growth and survival in cancer cells [153]. Deletion of the *Ptgs1* and *Ptgs2* genes coding for cyclooxygenase (COX) enzymes prevents the production of PGE_2_ and facilitates accumulation of cDC1s within the tumour [154]. In COX-deficient tumours, cDC1s form clusters at a distance from the tumour edge and blood vessels within the tumour, demonstrating more effective infiltration [155]. These PGE_2_-deficient tumours also had increased levels of natural killer (NK) cells, which localised in the same areas as cDC1s. NK cells recruit cDC1s to the tumour by highly expressing chemokines CCL5 and XCL1 [155]. Gene expression data sets and The Cancer Genome Atlas confirmed a similar relationship between NK cells and cDC1s in patients, and furthermore, higher intratumoral expression of NK cells and cDC1s positively correlates with survival in some cancers, including melanoma [155]. Since COX activity can be effectively blocked by existing drugs including aspirin, this mechanism of improving DC infiltration to tumours continues to be of huge interest for enhancing the effectiveness of immunotherapy.

In addition to suppressing immune responses by preventing immune cell recruitment, tumours are also able to control DC migration to tumour-draining lymph nodes. Cancer-associated vasculature has increased expression of the adhesion protein L1 [110]. Although its biological relevance in the tumour is not well understood, it was speculated that this may promote migration of non-antigen activated, immature DCs to the lymph node, where they can drive a tolerogenic response which supports tumour immune escape [110]. Alternatively, the tumour can inhibit DC migration to the tumour-draining lymph node by overexpressing transforming growth factor beta (TGF-β) [156–158], a cytokine found to be produced by many different cancers, and has been associated with poor outcome [159, 160]. TGF-β has previously been shown to inhibit the expression of CCR7 on BMDCs [161], suggesting that a lack of chemokine guidance may explain the decreased capacity of DCs to reach the tumour-draining lymph node. There have also been reports that TGF-β produced by the tumour may be able to travel to the tumour-draining lymph node and cause apoptosis of DCs [162]. A decreased number of DCs within the tumour-draining lymph node creates an immunosuppressive environment which may facilitate metastasis to these lymph nodes [157].

## Concluding remarks

**Fig. 1:**
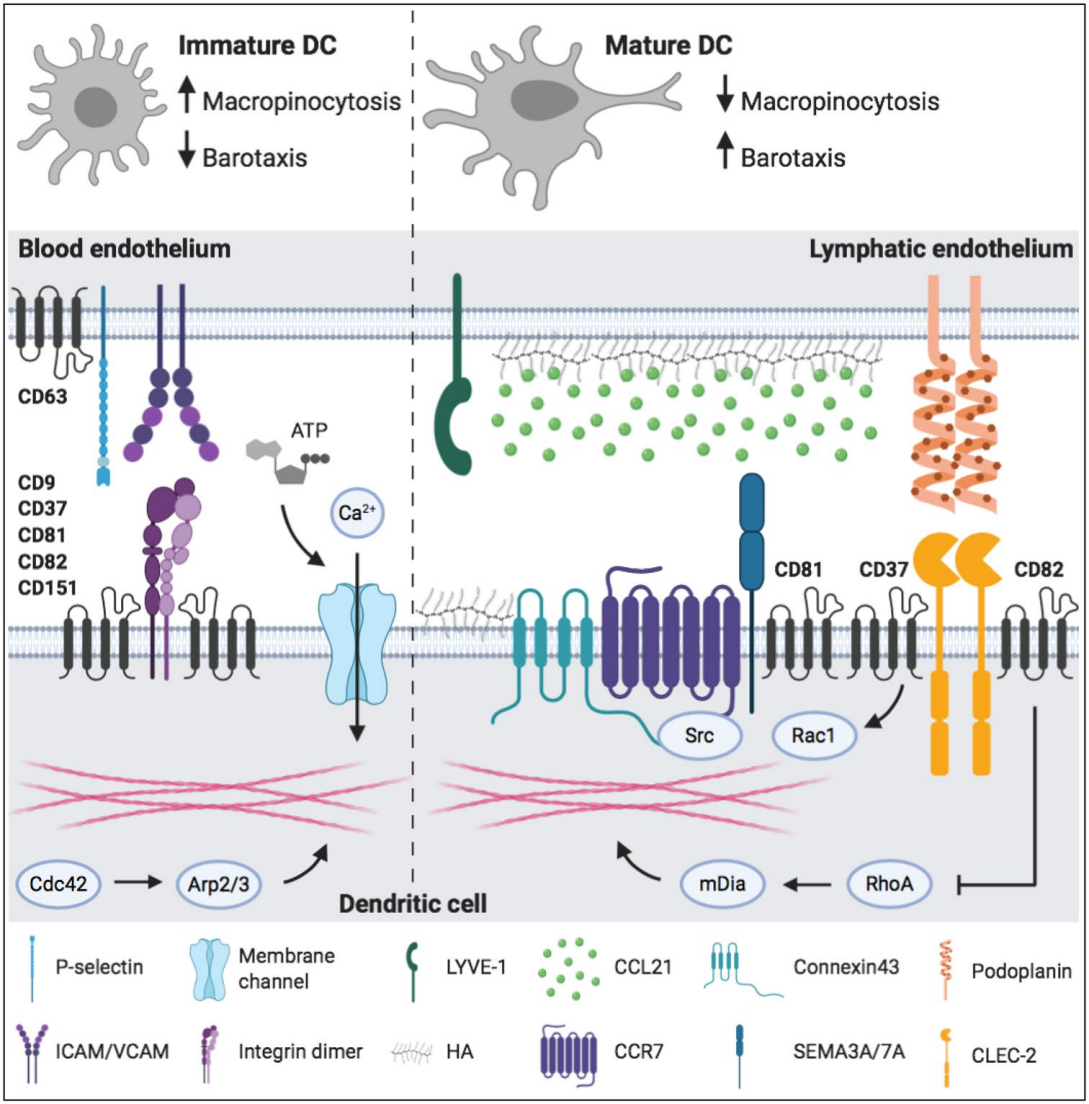
Surface proteins and cytoskeletal processes involved in dendritic cell migration. Left panel shows molecular mechanisms of precursor cells and immature DCs. Right panel shows molecular mechanisms driving directional migration of mature DCs. Tetraspanins are depicted as black four-transmembrane proteins. For detailed explanation, see the body of the text. *CCL21* chemokine ligand 21, *CCR7* chemokine receptor 7, *CLEC-2* C-type lectin-like receptor 2, *DC* dendritic cell, *HA* hyaluronic acid, *SEMA* semaphorin. Image created with BioRender.com

As sentinels of the immune system and bridge between the innate and adaptive immune system, DCs migrate through different tissues and across many barriers. DCs are equipped with a molecular toolbox to adapt to these different environments (Fig. 1). Environmental cues and cell–cell interactions result in integrin activation, increased expression of chemokine and C-type lectin receptors, and changes to the actin cytoskeleton. One mechanism crucial for the migration of activated DCs is the interaction between the chemokine CCL21 and its receptor CCR7 located on the surface of DCs [88, 91]. Although other molecular processes contribute to DC migration, none of them result in DC arrival at the lymph node without chemokine guidance. Except CCR7 [88, 91], deletion of other proteins did not completely inhibit DC migration, suggesting that proteins work synergistically to enable efficient and fast migration, but that on their own, they are not essential for DC migration to the lymph nodes.

Cancer cells often have immune escape mechanisms, which prevents the development of a successful anti-tumour immune response. The capability of DCs to present antigen to and activate T cells makes them essential for T cell-mediated tumour rejection [13, 14]. As such, modulation of DC function is of emerging interest to improve anti-cancer immunotherapy [147]. Most research has been focused on improving activation of DCs using tumour antigens or TLR ligands, and administration of ex vivo activated DCs, the so-called DC vaccines [147]. However, these strategies do not take into account the migratory capacity of DCs. Inhibition or reduction of TFG-β1 in tumours could potentially be used to increase migration of DCs to the tumour-draining lymph node to present tumour antigens [157, 158]. Furthermore, dampening PGE_2_ in the tumour microenvironment may be a novel strategy to increase the recruitment of DCs to the tumour [155]. This has particular relevance to a recently published study which indicated that intratumoral DCs may play a role in the efficacy of anti-tumour responses in anti-PD-1 therapy, which is already used in practice [163].

Tetraspanins control several aspects of anti-tumour immunity [164], but their role in DC migration from the tumour to the lymph nodes and vice versa has not been extensively addressed. One study reported increased tumour growth in *Cd37* knock-out mice [133]. This was caused by an impaired T cell-driven anti-tumour immune response due to migration failure of CD37-deficient DCs. Further research on the role of tetraspanins in controlling protein expression and DC signalling may enable the discovery of therapeutic strategies targeting tetraspanins to promote DC migration [164]. In conclusion, studies modulating DC migration in cancer are necessary to determine if this strategy, potentially in combination with current therapies, will improve anti-cancer immunity.

## References

[1] Steinman RM, Cohn ZA (1973). Identification of a novel cell type in peripheral lymphoid organs of mice: I. Morphology, quantitation, tissue distribution. J Exp Med.

[2] Nobel Media AB 2014 (2011) Ralph M. Steinman—facts. In: Nobelprize.org

[3] Schraml BU, van Blijswijk J, Zelenay S (2013). Genetic tracing via DNGR-1 expression history defines dendritic cells as a hematopoietic lineage. Cell.

[4] Collin M, Bigley V (2018). Human dendritic cell subsets: an update. Immunology.

[5] Reynolds G, Haniffa M (2015). Human and mouse mononuclear phagocyte networks: a tale of two species?. Front Immunol.

[6] Villani AC, Satija R, Reynolds G (2017). Single-cell RNA-seq reveals new types of human blood dendritic cells, monocytes, and progenitors. Science (80-).

[7] Alcántara-Hernández M, Leylek R, Wagar LE (2017). High-dimensional phenotypic mapping of human dendritic cells reveals interindividual variation and tissue specialization. Immunity.

[8] Elpek KG, Bellemare-Pelletier A, Malhotra D (2011). Lymphoid organ-resident dendritic cells exhibit unique transcriptional fingerprints based on subset and site. PLoS ONE.

[9] Worbs T, Hammerschmidt SI, Förster R (2017). Dendritic cell migration in health and disease. Nat Rev Immunol.

[10] Schulz O, Reis e Sousa C (2002). Cross-presentation of cell-associated antigens by CD8alpha+ dendritic cells is attributable to their ability to internalize dead cells. Immunology.

[11] Germain RN (1994). MHC-dependent antigen processing and peptide presentation: providing ligands for T lymphocyte activation. Cell.

[12] Matzinger P (2002). The danger model: a renewed sense of self. Science.

[13] Flamand V, Sornasse T, Thielemans K (1994). Murine dendritic cells pulsed in vitro with tumor antigen induce tumor resistance in vivo. Eur J Immunol.

[14] Fields RC, Shimizu K, Mulé JJ (1998). Murine dendritic cells pulsed with whole tumor lysates mediate potent antitumor immune responses in vitro and in vivo. Proc Natl Acad Sci USA.

[15] Coulie PG, Van den Eynde BJ, van der Bruggen P, Boon T (2014). Tumour antigens recognized by T lymphocytes: at the core of cancer immunotherapy. Nat Rev Cancer.

[16] Granucci F, Ferrero E, Foti M (1999). Early events in dendritic cell maturation induced by LPS. Microbes Infect.

[17] Larsen CP, Steinman RM, Witmer-Pack M (1990). Migration and maturation of Langerhans cells in skin transplants and explants. J Exp Med.

[18] Bousso P, Robey E (2003). Dynamics of CD8+ T cell priming by dendritic cells in intact lymph nodes. Nat Immunol.

[19] Ingulli E, Mondino A, Khoruts A, Jenkins MK (1997). In vivo detection of dendritic cell antigen presentation to CD4(+) T cells. J Exp Med.

[20] Levy S, Shoham T (2005). The tetraspanin web modulates immune-signalling complexes. Nat Rev Immunol.

[21] van Deventer SJ, Dunlock VME, van Spriel AB (2017). Molecular interactions shaping the tetraspanin web. Biochem Soc Trans.

[22] Termini CM, Gillette JM (2017). Tetraspanins function as regulators of cellular signaling. Front Cell Dev Biol.

[23] de Winde CM, Zuidscherwoude M, Vasaturo A (2015). Multispectral imaging reveals the tissue distribution of tetraspanins in human lymphoid organs. Histochem Cell Biol.

[24] Figdor CG, van Spriel AB (2009). Fungal pattern-recognition receptors and tetraspanins: partners on antigen-presenting cells. Trends Immunol.

[25] Jiang X, Zhang J, Huang Y (2015). Tetraspanins in cell migration. Cell Adhes Migr.

[26] Saiz ML, Rocha-Perugini V, Sánchez-Madrid F (2018). Tetraspanins as organizers of antigen-presenting cell function. Front Immunol.

[27] Yeung L, Hickey MJ, Wright MD (2018). The many and varied roles of tetraspanins in immune cell recruitment and migration. Front Immunol.

[28] O’Doherty U, Peng M, Gezelter S (1994). Human blood contains two subsets of dendritic cells, one immunologically mature and the other immature. Immunology.

[29] Geissmann F, Jung S, Littman DR (2003). Blood monocytes consist of two principal subsets with distinct migratory properties. Immunity.

[30] Ginhoux F, Liu K, Helft J (2009). The origin and development of nonlymphoid tissue CD103+ DCs. J Exp Med.

[31] Liu K, Victora GD, Schwickert TA (2009). In vivo analysis of dendritic cell development and homeostasis. Science (80-).

[32] Springer TA (1994). Traffic signals for lymphocyte recirculation and leukocyte emigration: the multistep paradigm. Cell.

[33] Pendl GG, Robert C, Steinert M (2002). Immature mouse dendritic cells enter inflamed tissue, a process that requires E- and P-selectin, but not P-selectin glycoprotein ligand 1. Blood.

[34] Alvarez D, Vollmann EH, von Andrian UH (2008). Mechanisms and consequences of dendritic cell migration. Immunity.

[35] Tedder TF, Steeber DA, Chen A, Engel P (1995). The selecting: vascular adhesion molecules. FASEB J.

[36] Arbonés ML, Ord DC, Ley K (1994). Lymphocyte homing and leukocyte rolling and migration are impaired in L-selectin-deficient mice. Immunity.

[37] Mayadas TN, Johnson RC, Rayburn H (1993). Leukocyte rolling and extravasation are severely compromised in P selectin-deficient mice. Cell.

[38] Doyle EL, Ridger V, Ferraro F (2011). CD63 is an essential cofactor to leukocyte recruitment by endothelial P-selectin. Blood.

[39] Auffray C, Fogg D, Garfa M (2007). Monitoring of blood vessels and tissues by a population of monocytes with patrolling behavior. Science.

[40] Shaw AR, Domanska A, Mak A (1995). Ectopic expression of human and feline CD9 in a human B cell line confers beta 1 integrin-dependent motility on fibronectin and laminin substrates and enhanced tyrosine phosphorylation. J Biol Chem.

[41] Reyes R, Monjas A, Yánez-Mó M (2015). Different states of integrin LFA-1 aggregation are controlled through its association with tetraspanin CD9. Biochim Biophys Acta Mol Cell Res.

[42] van Spriel AB, de Keijzer S, van der Schaaf A (2012). The tetraspanin CD37 orchestrates the α(4)β(1) integrin-Akt signaling axis and supports long-lived plasma cell survival. Sci Signal.

[43] Feigelson SW, Grabovsky V, Shamri R (2003). The CD81 tetraspanin facilitates instantaneous leukocyte VLA-4 adhesion strengthening to vascular cell adhesion molecule 1 (VCAM-1) under shear flow. J Biol Chem.

[44] Karamatic Crew V, Burton N, Kagan A (2004). CD151, the first member of the tetraspanin (TM4) superfamily detected on erythrocytes, is essential for the correct assembly of human basement membranes in kidney and skin. Blood.

[45] Mannion BA, Berditchevski F, Kraeft SK (1996). Transmembrane-4 superfamily proteins CD81 (TAPA-1), CD82, CD63, and CD53 specifically associated with integrin alpha 4 beta 1 (CD49d/CD29). J Immunol.

[46] Franz J, Brinkmann BF, Konig M (2016). Nanoscale imaging reveals a tetraspanin-CD9 coordinated elevation of endothelial ICAM-1 clusters. PLoS ONE.

[47] Wee JL, Schulze KE, Jones EL (2015). Tetraspanin CD37 regulates Beta2 integrin-mediated adhesion and migration in neutrophils. J Immunol.

[48] Shibagaki N, Hanada KI, Yamashita H (1999). Overexpression of CD82 on human T cells enhances LFA-1/ICAM-1-mediated cell-cell adhesion: functional association between CD82 and LFA-1 in T cell activation. Eur J Immunol.

[49] Dunlock VE (2020). Tetraspanin CD53: an overlooked regulator of immune cell function. Med Microbiol Immunol.

[50] Knobeloch KP, Wright MD, Ochsenbein AF (2000). Targeted inactivation of the tetraspanin CD37 impairs T-cell-dependent B-cell response under suboptimal costimulatory conditions. Mol Cell Biol.

[51] Maecker HT, Levy S (1997). Normal lymphocyte development but delayed humoral immune response in CD81-null mice. J Exp Med.

[52] Charrin S, le Naour F, Silvie O (2009). Lateral organization of membrane proteins: tetraspanins spin their web. Biochem J.

[53] Zelenay S, Reis e Sousa C (2013). Adaptive immunity after cell death. Trends Immunol.

[54] Janeway CA, Medzhitov R (2002). Innate immune recognition. Annu Rev Immunol.

[55] Mogensen TH (2009). Pathogen recognition and inflammatory signaling in innate immune defenses. Clin Microbiol Rev.

[56] Krysko O, Løve Aaes T, Bachert C (2013). Many faces of DAMPs in cancer therapy. Cell Death Dis.

[57] Akira S, Uematsu S, Takeuchi O (2006). Pathogen recognition and innate immunity. Cell.

[58] Kawai T, Akira S (2007). Signaling to NF-κB by Toll-like receptors. Trends Mol Med.

[59] Hayden MS, West AP, Ghosh S (2006). NF-κB and the immune response. Oncogene.

[60] Höpken UE, Foss HD, Meyer D (2002). Up-regulation of the chemokine receptor CCR7 in classical but not in lymphocyte-predominant Hodgkin disease correlates with distinct dissemination of neoplastic cells in lymphoid organs. Blood.

[61] Trevejo JM, Marino MW, Philpott N (2001). TNF-α-dependent maturation of local dendritic cells is critical for activating the adaptive immune response to virus infection. Proc Natl Acad Sci USA.

[62] Gallucci S, Lolkema M, Matzinger P (1999). Natural adjuvants: endogenous activators of dendritic cells. Nat Med.

[63] Nolte MA, Leibundgut-Landmann S, Joffre O, Reis e Sousa C (2007). Dendritic cell quiescence during systemic inflammation driven by LPS stimulation of radioresistant cells in vivo. J Exp Med.

[64] Sáez PJ, Vargas P, Shoji KF (2017). ATP promotes the fast migration of dendritic cells through the activity of pannexin 1 channels and P2X7 receptors. Sci Signal.

[65] Sallusto F, Cella M, Danieli C, Lanzavecchia A (1995). Dendritic cells use macropinocytosis and the mannose receptor to concentrate macromolecules in the major histocompatibility complex class II compartment: Downregulation by cytokines and bacterial products. J Exp Med.

[66] Reis e Sousa C, Stahl PD, Austyn JM (1993). Phagocytosis of antigens by Langerhans cells in vitro. J Exp Med.

[67] Banchereau J, Steinman RM (1998). Dendritic cells and the control of immunity. Nature.

[68] Deckers J, Hammad H, Hoste E (2018). Langerhans cells: sensing the environment in health and disease. Front Immunol.

[69] Nishibu A, Ward BR, Jester JV (2006). Behavioral responses of epidermal langerhans cells in situ to local pathological stimuli. J Invest Dermatol.

[70] Kissenpfennig A, Henri S, Dubois B (2005). Dynamics and function of langerhans cells in vivo: dermal dendritic cells colonize lymph node areas distinct from slower migrating langerhans cells. Immunity.

[71] Lämmermann T, Bader BL, Monkley SJ (2008). Rapid leukocyte migration by integrin-independent flowing and squeezing. Nature.

[72] de Bruyn PPH (1946). The amoeboid movement of the mammalian leukocyte in tissue culture. Anat Rec.

[73] Charras G, Paluch E (2008). Blebs lead the way: how to migrate without lamellipodia. Nat Rev Mol Cell Biol.

[74] Lämmermann T, Sixt M (2009). Mechanical modes of ‘amoeboid’ cell migration. Curr Opin Cell Biol.

[75] Renkawitz J, Schumann K, Weber M (2009). Adaptive force transmission in amoeboid cell migration. Nat Cell Biol.

[76] Balaban NQ, Schwarz US, Riveline D (2001). Force and focal adhesion assembly: a close relationship studied using elastic micropatterned substrates. Nat Cell Biol.

[77] Legant WR, Miller JS, Blakely BL (2010). Measurement of mechanical tractions exerted by cells in three-dimensional matrices. Nat Methods.

[78] Moreau HD, Blanch-Mercader C, Attia R (2019). Macropinocytosis overcomes directional bias in dendritic cells due to hydraulic resistance and facilitates space exploration. Dev Cell.

[79] Bergert M, Erzberger A, Desai RA (2015). Force transmission during adhesion-independent migration. Nat Cell Biol.

[80] Faure-André G, Vargas P, Yuseff MI (2008). Regulation of dendritic cell migration by CD74, the MHC class II-associated invariant chain. Science (80-).

[81] Chabaud M, Heuze ML, Bretou M (2015). Cell migration and antigen capture are antagonistic processes coupled by myosin II in dendritic cells. Nat Commun.

[82] Vargas P, Maiuri P, Bretou M (2016). Innate control of actin nucleation determines two distinct migration behaviours in dendritic cells. Nat Cell Biol.

[83] Rottner K, Schaks M (2019). Assembling actin filaments for protrusion. Curr Opin Cell Biol.

[84] Prentice-Mott HV, Chang CH, Mahadevan L (2013). Biased migration of confined neutrophil-like cells in asymmetric hydraulic environments. Proc Natl Acad Sci USA.

[85] McLellan AD, Starling GC, Williams LA (1995). Activation of human peripheral blood dendritic cells induces the CD86 co-stimulatory molecule. Eur J Immunol.

[86] Wieczorek M, Abualrous ET, Sticht J (2017). Major histocompatibility complex (MHC) class I and MHC class II proteins: conformational plasticity in antigen presentation. Front Immunol.

[87] Rock KL, Rothstein L, Gamble S, Fleischacker C (1993). Characterization of antigen-presenting cells that present exogenous antigens in association with class I MHC molecules. J Immunol.

[88] Sallusto F, Schaerli P, Loetscher P (1998). Rapid and coordinated switch in chemokine receptor expression during dendritic cell maturation. Eur J Immunol.

[89] Björck P, Leong HX, Engleman EG (2011). Plasmacytoid dendritic cell dichotomy: identification of IFN-α producing cells as a phenotypically and functionally distinct subset. J Immunol.

[90] Zhang H, Gregorio JD, Iwahori T (2017). A distinct subset of plasmacytoid dendritic cells induces activation and differentiation of B and T lymphocytes. Proc Natl Acad Sci.

[91] Förster R, Schubel A, Breitfeld D (1999). CCR7 coordinates the primary immune response by establishing functional microenvironments in secondary lymphoid organs. Cell.

[92] Weber M, Hauschild R, Schwarz J (2013). Interstitial dendritic cell guidance by haptotactic chemokine gradients. Science (80-).

[93] Britschgi MR, Favre S, Luther SA (2010). CCL21 is sufficient to mediate DC migration, maturation and function in the absence of CCL19. Eur J Immunol.

[94] Haessler U, Pisano M, Mingming Wu, Swartz MA (2011). Dendritic cell chemotaxis in 3D under defined chemokine gradients reveals differential response to ligands CCL21 and CCL19. Proc Natl Acad Sci USA.

[95] Johnson LA, Jackson DG (2010). Inflammation-induced secretion of CCL21 in lymphatic endothelium is a key regulator of integrin-mediated dendritic cell transmigration. Int Immunol.

[96] Petrie RJ, Doyle AD, Yamada KM (2009). Random versus directionally persistent cell migration. Nat Rev Mol Cell Biol.

[97] Hauser MA, Schaeuble K, Kindinger I (2016). Inflammation-induced CCR7 oligomers form scaffolds to integrate distinct signaling pathways for efficient cell migration. Immunity.

[98] Ruez R, Dubrot J, Zoso A (2018). Dendritic cell migration toward CCL21 gradient requires functional Cx43. Front Physiol.

[99] Sorgen PL, Duffy HS, Sahoo P (2004). Structural changes in the carboxyl terminus of the gap junction protein connexin43 indicates signaling between binding domains for c-Src and zonula occludens-1. J Biol Chem.

[100] van Rijn A, Paulis L, Te Riet J (2016). Semaphorin 7A promotes chemokine-driven dendritic cell migration. J Immunol.

[101] Baluk P, Fuxe J, Hashizume H (2007). Functionally specialized junctions between endothelial cells of lymphatic vessels. J Exp Med.

[102] Pflicke H, Sixt M (2009). Preformed portals facilitate dendritic cell entry into afferent lymphatic vessels. J Exp Med.

[103] Banerji S, Ni J, Wang SX (1999). LYVE-1, a new homologue of the CD44 glycoprotein, is a lymph-specific receptor for hyaluronan. J Cell Biol.

[104] Mummert ME, Mummert D, Edelbaum D (2002). Synthesis and surface expression of hyaluronan by dendritic cells and its potential role in antigen presentation. J Immunol.

[105] Johnson LA, Banerji S, Lawrance W (2017). Dendritic cells enter lymph vessels by hyaluronan-mediated docking to the endothelial receptor LYVE-1. Nat Immunol.

[106] Johnson LA, Clasper S, Holt AP (2006). An inflammation-induced mechanism for leukocyte transmigration across lymphatic vessel endothelium. J Exp Med.

[107] Vigl B, Aebischer D, Nitschké M (2011). Tissue inflammation modulates gene expression of lymphatic endothelial cells and dendritic cell migration in a stimulus-dependent manner. Blood.

[108] Maness PF, Schachner M (2007). Neural recognition molecules of the immunoglobulin superfamily: signaling transducers of axon guidance and neuronal migration. Nat Neurosci.

[109] Pancook JD, Reisfeld RA, Varki N (1997). Expression and regulation of the neural cell adhesion molecule L1 on human cells of myelomonocytic and lymphoid origin. J Immunol.

[110] Maddaluno L, Verbrugge SE, Martinoli C (2009). The adhesion molecule L1 regulates transendothelial migration and trafficking of dendritic cells. J Exp Med.

[111] Vaahtomeri K, Brown M, Hauschild R (2017). Locally triggered release of the chemokine CCL21 promotes dendritic cell transmigration across lymphatic endothelia. Cell Rep.

[112] Takamatsu H, Takegahara N, Nakagawa Y (2010). Semaphorins guide the entry of dendritic cells into the lymphatics by activating myosin II. Nat Immunol.

[113] Russo E, Teijeira A, Vaahtomeri K (2016). Intralymphatic CCL21 promotes tissue egress of dendritic cells through afferent lymphatic vessels. Cell Rep.

[114] Tal O, Lim HY, Gurevich I (2011). DC mobilization from the skin requires docking to immobilized CCL21 on lymphatic endothelium and intralymphatic crawling. J Exp Med.

[115] Smith A, Bracke M, Leitinger B (2003). LFA-1-induced T cell migration on ICAM-1 involves regulation of MLCK-mediated attachment and ROCK-dependent detachment. J Cell Sci.

[116] Soriano SF, Hons M, Schumann K (2011). In vivo analysis of uropod function during physiological T cell trafficking. J Immunol.

[117] Nitschké M, Aebischer D, Abadier M (2012). Differential requirement for ROCK in dendritic cell migration within lymphatic capillaries in steady-state and inflammation. Blood.

[118] Ikomi F, Kawai Y, Ohhashi T (2012). Recent advance in lymph dynamic analysis in lymphatics and lymph nodes. Ann Vasc Dis.

[119] Braun A, Worbs T, Moschovakis GL (2011). Afferent lymph–derived T cells and DCs use different chemokine receptor CCR7–dependent routes for entry into the lymph node and intranodal migration. Nat Immunol.

[120] Kaldjian EP, Gretz JE, Anderson AO (2001). Spatial and molecular organization of lymph node T cell cortex: a labyrinthine cavity bounded by an epithelium-like monolayer of fibroblastic reticular cells anchored to basement membrane-like extracellular matrix. Int Immunol.

[121] Willard-Mack CL (2006). Normal structure, function, and histology of lymph nodes. Toxicol Pathol.

[122] Fletcher AL, Acton SE, Knoblich K (2015). Lymph node fibroblastic reticular cells in health and disease. Nat Rev Immunol.

[123] Link A, Vogt TK, Favre S (2007). Fibroblastic reticular cells in lymph nodes regulate the homeostasis of naive T cells. Nat Immunol.

[124] Peduto L, Dulauroy S, Lochner M (2009). Inflammation recapitulates the ontogeny of lymphoid stromal cells. J Immunol.

[125] Mourão-Sá D, Robinson MJ, Zelenay S (2011). CLEC-2 signaling via Syk in myeloid cells can regulate inflammatory responses. Eur J Immunol.

[126] Acton SE, Astarita JL, Malhotra D (2012). Podoplanin-rich stromal networks induce dendritic cell motility via activation of the C-type lectin receptor CLEC-2. Immunity.

[127] de Winde CM, Matthews AL, van Deventer S (2018). C-type lectin-like receptor 2 (CLEC-2)-dependent DC migration is controlled by tetraspanin CD37. J Cell Sci.

[128] Kerjaschki D (2004). Lymphatic neoangiogenesis in human kidney transplants is associated with immunologically active lymphocytic infiltrates. J Am Soc Nephrol.

[129] Nakazawa Y, Sato S, Naito M (2008). Tetraspanin family member CD9 inhibits Aggrus/podoplanin-induced platelet aggregation and suppresses pulmonary metastasis. Blood.

[130] de Winde CM, Makris S, Millward L et al (2019) Podoplanin function is switched by partner proteins on fibroblastic reticular cells. bioRxiv 793141. 10.1101/793141

[131] Bergsma A, Ganguly SS, Wiegand ME (2019). Regulation of cytoskeleton and adhesion signaling in osteoclasts by tetraspanin CD82. Bone Rep.

[132] Suzuki-Inoue K, Fuller GLJ, García A (2006). A novel Syk-dependent mechanism of platelet activation by the C-type lectin receptor CLEC-2. Blood.

[133] Gartlan KH, Wee JL, Demaria MC (2013). Tetraspanin CD37 contributes to the initiation of cellular immunity by promoting dendritic cell migration. Eur J Immunol.

[134] Tejera E, Rocha-Perugini V, López-Martín S (2013). CD81 regulates cell migration through its association with Rac GTPase. Mol Biol Cell.

[135] Quast T, Eppler F, Semmling V (2011). CD81 is essential for the formation of membrane protrusions and regulates Rac1-activation in adhesion-dependent immune cell migration. Blood.

[136] Jones EL, Wee JL, Demaria MC (2016). Dendritic cell migration and antigen presentation are coordinated by the opposing functions of the tetraspanins CD82 and CD37. J Immunol.

[137] Hanahan D, Weinberg RA (2000). The hallmarks of cancer. Cell.

[138] Hanahan D, Weinberg RA (2011). Hallmarks of cancer: the next generation. Cell.

[139] Balkwill FR, Capasso M, Hagemann T (2012). The tumor microenvironment at a glance. J Cell Sci.

[140] Swiecki M, Colonna M (2015). The multifaceted biology of plasmacytoid dendritic cells. Nat Rev Immunol.

[141] Conrad C, Gregorio J, Wang Y-H (2012). Plasmacytoid dendritic cells promote immunosuppression in ovarian cancer via ICOS costimulation of Foxp3+ T-regulatory cells. Cancer Res.

[142] Faget J, Sisirak V, Blay J-Y (2013). ICOS is associated with poor prognosis in breast cancer as it promotes the amplification of immunosuppressive CD4 ^+^ T cells by plasmacytoid dendritic cells. Oncoimmunology.

[143] Tel J, Aarntzen EHJG, Baba T (2013). Natural human plasmacytoid dendritic cells induce antigen-specific T-cell responses in melanoma patients. Cancer Res.

[144] van Beek JJP, Flórez-Grau G, Gorris MAJ (2020). Human pDCs are superior to cDC2s in attracting cytolytic lymphocytes in melanoma patients receiving DC vaccination. Cell Rep.

[145] Kranz LM, Diken M, Haas H (2016). Systemic RNA delivery to dendritic cells exploits antiviral defence for cancer immunotherapy. Nature.

[146] Perez CR, De Palma M (2019). Engineering dendritic cell vaccines to improve cancer immunotherapy. Nat Commun.

[147] Wculek SK, Cueto FJ, Mujal AM (2019). Dendritic cells in cancer immunology and immunotherapy. Nat Rev Immunol.

[148] Böttcher JP, Reis e Sousa C (2018). The role of type 1 conventional dendritic cells in cancer immunity. Trends Cancer.

[149] Roberts EW, Broz ML, Binnewies M (2016). Critical role for CD103+/CD141+ dendritic cells bearing CCR7 for tumor antigen trafficking and priming of T cell immunity in melanoma. Cancer Cell.

[150] Valenzuela J, Schmidt C, Mescher M (2002). The roles of IL-12 in providing a third signal for clonal expansion of naive CD8 T cells. J Immunol.

[151] Spranger S, Dai D, Horton B, Gajewski TF (2017). Tumor-residing Batf3 dendritic cells are required for effector T cell trafficking and adoptive T cell therapy. Cancer Cell.

[152] Hangai S, Ao T, Kimura Y (2016). PGE2 induced in and released by dying cells functions as an inhibitory DAMP. Proc Natl Acad Sci USA.

[153] Wang D, DuBois RN (2010). Eicosanoids and cancer. Nat Rev Cancer.

[154] Zelenay S, van der Veen AG, Böttcher JP (2015). Cyclooxygenase-dependent tumor growth through evasion of immunity. Cell.

[155] Böttcher JP, Bonavita E, Chakravarty P (2018). NK cells stimulate recruitment of cDC1 into the tumor microenvironment promoting cancer immune control. Cell.

[156] Halliday GM, Le S (2001). Transforming growth factor-β produced by progressor tumors inhibits, while IL-10 produced by regressor tumors enhances, Langerhans cell migration from skin. Int Immunol.

[157] Imai K, Minamiya Y, Koyota S (2012). Inhibition of dendritic cell migration by transforming growth factor-β1 increases tumor-draining lymph node metastasis. J Exp Clin Cancer Res.

[158] Weber F, Byrne SN, Le S (2005). Transforming growth factor-β1 immobilises dendritic cells within skin tumours and facilitates tumour escape from the immune system. Cancer Immunol Immunother.

[159] Saito H, Tsujitani S, Oka S (1999). The expression of transforming growth factor-β1 is significantly correlated with the expression of vascular endothelial growth factor and poor prognosis of patients with advanced gastric carcinoma. Cancer.

[160] Hasegawa Y, Takanashi S, Kanehira Y (2001). Transforming growth factor-β1 level correlates with angiogenesis, tumor progression, and prognosis in patients with nonsmall cell lung carcinoma. Cancer.

[161] Ogata M, Zhang Y, Wang Y (1999). Chemotactic response toward chemokines and its regulation by transforming growth factor-β1 of murine bone marrow hematopoietic progenitor cell-derived different subset of dendritic cells. Blood.

[162] Ito M, Minamiya Y, Kawai H (2006). Tumor-derived TGFβ-1 induces dendritic cell apoptosis in the sentinel lymph node. J Immunol.

[163] Garris CS, Arlauckas SP, Kohler RH (2018). Successful anti-PD-1 cancer immunotherapy requires T cell-dendritic cell crosstalk involving the cytokines IFN-γ and IL-12. Immunity.

[164] Schaper F, van Spriel AB (2018). Antitumor immunity is controlled by tetraspanin proteins. Front Immunol.

[165] Granot T, Senda T, Carpenter DJ (2017). Dendritic cells display subset and tissue-specific maturation dynamics over human life. Immunity.

[166] Zuidscherwoude M, Worah K, van der Schaaf A (2017). Differential expression of tetraspanin superfamily members in dendritic cell subsets. PLoS ONE.

[167] Balkow S, Heinz S, Schmidbauer P (2010). LFA-1 activity state on dendritic cells regulates contact duration with T cells and promotes T-cell priming. Blood.

[168] Aerts-Toegaert C, Heirman C, Tuyaerts S (2007). CD83 expression on dendritic cells and T cells: correlation with effective immune responses. Eur J Immunol.

[169] Ma DY, Clark EA (2009). The role of CD40 and CD154/CD40L in dendritic cells. Semin Immunol.

[170] Cao L, Yoshino T, Kawasaki N (1997). Anti-CD53 monoclonal antibody induced LFA-1/ICAM-1-dependent and -independent lymphocyte homotypic cell aggregation. Immunobiology.

[171] Todros-Dawda I, Kveberg L, Vaage JT, Inngjerdingen M (2014). The tetraspanin CD53 modulates responses from activating NK cell receptors, promoting LFA-1 activation and dampening NK cell effector functions. PLoS ONE.

[172] VanCompernolle SE, Levy S, Todd SC (2001). Anti-CD81 activates LFA-1 on T cells and promotes T cell-B cell collaboration. Eur J Immunol.

[173] Szöllósi J, Horejsí V, Bene L (1996). Supramolecular complexes of MHC class I, MHC class II, CD20, and tetraspan molecules (CD53, CD81, and CD82) at the surface of a B cell line JY. J Immunol.

[174] Lagaudrière-Gesbert C, Lebel-Binay S, Wiertz E (1997). The tetraspanin protein CD82 associates with both free HLA class I heavy chain and heterodimeric beta 2-microglobulin complexes. J Immunol.

[175] Angelisova P, Hilgert I, Horejsi V (1994). Association of four antigens of the tetraspans family (CD37, CD53, TAPA-1, and R2/C33) with MHC class II glycoproteins. Immunogenetics.

[176] Rubinstein E, Le Naour F, Lagaudriere-Gesbert C (1996). CD9, CD63, CD81, and CD82 are components of a surface tetraspan network connected to HLA-DR and VLA integrins. Eur J Immunol.

[177] Engering A, Pieters J (2001). Association of distinct tetraspanins with MHC class II molecules at different subcellular locations in human immature dendritic cells. Int Immunol.

[178] Zuidscherwoude M, Göttfert F, Dunlock VME (2015). The tetraspanin web revisited by super-resolution microscopy. Sci Rep.

[179] Sheng K-CC, van Spriel AB, Gartlan KH (2009). Tetraspanins CD37 and CD151 differentially regulate Ag presentation and T-cell co-stimulation by DC. Eur J Immunol.

